# Validation of the Farsi version of the medical outcomes study-social support survey for mammography

**DOI:** 10.1186/s12889-018-6174-2

**Published:** 2018-11-20

**Authors:** Maryam Khazaee-Pool, Mitra Bahrami, John S. Luque, Tahereh Pashaei, Parvaneh Taymoori, Deam Roshani

**Affiliations:** 1 Department of Health Education and Promotion, School of Public Health, Zanjan University of Medical Sciences, Zanjan, Iran; 2 Health Education and Promotion, Student Research Committee, Kurdistan University of Medical Sciences, Sanandaj, Iran; 3 Institute of Public Health, Florida A&M University, Science Research Center, Tallahassee, Florida USA; 4 Environmental Health Research Center, Research Institute for Health Development, Kurdistan University of Medical Sciences, Sanandaj, Iran; 5 Faculty of Medicine, Social Determinants of Health Research Center, Research Institute for Health Development, Kurdistan University of Medical Sciences, Sanandaj, Iran

**Keywords:** Validation, Farsi version, Mammography, Social support

## Abstract

**Background:**

Social support can provide psychosocial benefits to promote positive health behaviors such as mammography screening. The purpose of this study was to assess the psychometric properties of the Mammography Social Support (MSS) scale among Iranian woman.

**Methods:**

Participants were selected from women referring to healthcare centers in Sanandaj, Iran. A total of 434 questionnaires were completed (response rate 91%). The study sample for study 1 included 204 participants for the Exploratory Factor Analysis (EFA). Construct validity was determined by confirmatory factor analysis (CFA) using a study sample of 230 women in study 2. The reliability coefficient for each scale was calculated using Cronbach’s alpha, corrected item-total correlations and test-retest respectively.

**Results:**

CFA affirmed the three-factor structure of the MSS in measuring the functional dimensions of social support for mammography behavior consisting of 19 items. Initial results of the CFA did not fully support the proposed three-factor model. After the model was modified, the fit indices indicated, *x*^2^ was 2.3, Comparative Fit Index (CFI) = 0.96, Tucker- Lewis Index (TLI) = 0.95 providing a strong fit to the data. Cronbach’s alphas for the subscales ranged from 0.82 and 0.90, whereas the alpha for the overall scale was 0.91. The 2-week test-retest reliability of MSS was 0.95.

**Conclusion:**

This study provides evidence for the psychometric properties to support the Farsi version of the MSS when applied to Iranian women. Exploring the three-factor model in relation to related concepts is suggested for future studies.

## Background

In many countries screening for breast cancer has been accomplished with the intent of detecting breast cancer at the beginning stages and thereby affording an improved prognosis. Early detection is one of the most important strategies for reducing breast cancer mortality followed by treatment for early stage cancer [1]. According to national data in Iran, a significant number of women (80%) have never obtained a mammogram or are not adherent (73%) with screening guidelines [2, 3]. Factors influencing participation with mammography screening among Iranian women reported in previous studies include perceived severity, perceived susceptibility [4–6], higher perceived barriers, and self-efficacy related to receipt of mammography screening [7]. Integration of subjective norms and perceived behavioral control with breast cancer screening interventions may increase participation in mammography screening among Iranian women [8]. Greater perceived social support predicted more repeat mammography behavior among Iranian women than with lower social support scores [3].

The concept of social support was first presented in the 1970s [9]. Results of a comprehensive literature review showed social support is described in terms of its type, function, and source [10]. In the literature, several definitions have been proposed for social support [11]. Ben- David, and Leichtentritt in their definition of social support focused on the level of social support in meeting the needs of individuals through interaction with others [11]. Caplan’s definition of social support invoked the necessity of providing counseling [12]. Among these definitions, the sources of social support refer to the sources of that support as described by the research of Zimet, Dahlem [13], Zimet, Farley [14], and Sherbourne & Stewart [15]. In addition, there are supporting experimental data to demonstrate the relationship between diverse types of social support and influence on different health outcomes [15, 16]. Sherbourne differentiated between (a) tangible support, which includes a provision of material help; (b) informational support, which offers a personalized direction and feedback; (c) emotional support, which expresses empathetic understanding; (d) positive social interaction, which offers companionship; and (e) affectionate support, which conveys love and affection [15].

The link between social support and breast cancer screening [mammography, breast self-exam (BSE), or clinical breast exam (CBE)] is unclear because findings from previous studies have not been consistent. Some of the inconsistent findings from these studies may be explained by the use of different measurement scales of social support and choosing different screening outcomes (mammography, BSE, or CBE). For example, Jensen [17] reported that low social support (eight items) was associated with non-participation in breast cancer screening. Documet [18] found that mammography receipt was notably influenced by social support in a US study (OR = 1.43). Their study assessed the emotional/informational, tangible, affectionate support and positive social interactions using a four-item scale [19]. Silav and colleagues [20] reported that global social support and emotional/informational and Positive Social Interaction (PSI) were associated with BSE in a Brazilian study. A US study by Messina that measured social support using six items reported that emotional/informational support and PSI, but not instrumental/affection support, were significantly associated with having regular mammography screening, BSE, and CBE [21]. The results of a Brazilian study by Andrade and colleagues suggested the highest scores in the five aspects of social support (material, emotional, affective, information, and PSI) related with a higher frequency of BSE [22]. They used a 19-item scale with five dimensions of social support questions on social networks and support [23]. The results of an Argentine study by Gamarra and colleagues did not find an association between social support and BSE or CBE, using a scale which contained 11 items on two dimensions, emotional and affective support [24]. Katapodi examined the association between social support and BSE as well mammography in a multicultural community using a social support scale with 5 items [25]. The results identified group differences on the scores of social support among three groups stratified by mammography status; never, once or twice a lifetime, or every one to two years. The lower perceived social support was associated with lower adherence to screening guidelines (for BSE or CBE). There were major differences on average social support scores between those rarely practiced BSE and those who did not practice BSE according to recommended screening guidelines.

### Measurement of perceived social support

Social support is generally conceptualized by different categories. Among these three categories of social support: (a) social embeddedness [26], (b) enacted support, [27] and (c) perceived social support [28], the highest endorsement has been given to the measurement of perceived social support [29]. In measuring social support, an emphasis has been placed on evaluating the multi-dimensional functions of the individual’s support system [15, 30]. Among the available scales that measure the functional features of perceived social support [31–33], it was noted there were some limitations for these measures, for example, no evidence of content validity for use in a specific target population, or an insufficient assessment of the diverse forms of functional social support [31].

These challenges in measuring social support led to the development and testing of the Medical Outcomes Study Social Support Survey (MOS-SSS) by Sherbourne & Stewart (MOS-SSS) by Sherbourne & Stewart [15]. It contains 19 functional support items and included four subscales: tangible, emotional and informational, affectionate support, and positive social interaction. Tangible support contains 4 items measuring the offering of material aid or behavioral assistance. Emotional support contains 4 items: measuring the expression of positive affect, empathetic understanding, and expressions of encouragement and feelings. Informational support includes 4 items for assessing the provisions of advice, information, guidance or feedback. Affectionate support contains 3 items measuring the expressions of love and affection. Positive social interaction is measured by 4 items related to the access of other persons to engage in enjoyable activities. Participants were asked to answer the questions using a 5-point Likert measure ranged 1 = “none of the time” to 5 = “all of the time” (5). The results supported the reliability of MOS-SSS (Cronbach’s alpha of 0.97 for the overall scale and 0.91–0.96 for each of the four subscales) [15].

In terms of measuring social support in the breast cancer screening context [17, 20, 22, 24], and in mammography specifically [18, 21, 25], it should be noted that most social support measures were not purposely developed for use in these types of screening contexts. Some researchers have studied the potential importance of social support and breast cancer screening practices, [17, 18, 24, 34–37] but none of the studies used a psychometrically validated and tested social support instrument specifically tested for mammography screening [3, 18, 21, 25]. Another measurement limitation is an insufficient measurement tool to differentiate types of functional social support [17]. For instance, repeat mammography behavior was greater among Iranian women with higher emotional/informational, tangible and affectionate/positive social support scores than women with lower social support scores [3]. To comprehensively assess the relationship between social support and mammography behavior, a validated instrument for providing clear conceptual and operational definitions was needed. It is expected the assessment of perceived social support might provide the information linked to the influence of an individual’s support system with respect to mammography adherence.

While other researchers have studied the potential importance of social support and breast cancer screening practices, (10–16) none of studies used a psychometrically validated and tested social support instrument specifically tested for mammography screening. This study aimed to evaluate the psychometric properties of a scale of perceived social support for mammography screening among Iranian women. This is the first study to evaluate the psychometric properties of the Mammography Social Support (MSS) scale in this population.

### Methods instrument

The inclusion of evaluation of support from significant others makes it particularly relevant in the context of breast cancer screening behaviors. The MOS-SSS is one of the most commonly used self-report instruments to measure social support. It has been tested in different contexts [38, 39] including in repeat mammography among Iranian women [3], in different languages [40–43] and with women from different cultural backgrounds [10, 44], the MOS-SSS has been adapted to measure social support for use in the Iranian population; however, the MOS-SSS has not been validated in Iranian society. The questionnaires may be affected by the context in which they are used and differences in culture. So, the research team used a back-translation method to produce a linguistically equivalent instrument in Farsi [45]. This included first translating the draft into Farsi. A bilingual professional translator who was a master’s prepared health promotion expert translated the English version of the MOS-SSS to Farsi. The equivalence between the original English version and the Farsi version of the MOS-SSS was evaluated by a second bilingual translator. In the case of differences between the original and back-translated English versions, the investigators identified the causes for the discrepancies and resolved all items.

### Content validity

The draft Farsi version of the MOS-SSS was also evaluated by an expert panel. The relevance and appropriateness of items to Iranian culture were reviewed by three professors in health education, two psychologists, one midwife and two oncology nurses. The experts evaluated the items’ Content Validity Index)CVI(and assessed each item on a four-point scale: 4 = “very relevant,” 3 = “relevant with some adjustment to phrasing,” 2 = “only relevant if the phrasing is profoundly adjusted,” and 1 = “irrelevant.” Experts provided improvements in wording for each item. In the case of an expert panel member rating any item less than 4, then they were asked to provide recommendations for changing or deleting the item. We defined the CVI scale’s score of greater than 0.79 as the criteria for confirmation of content validity based on suggestions from the World Health Organization [45]. Experts considered the remaining 19 items in the instrument. According to the experts’ feedback, some modifications in wording were made to tailor the questionnaire and the relationship between social support and mammography screening as follows: Item 2, “Someone to give you information to help you understand a situation” was changed to “Someone to give you information to help you understand the mammogram procedure.” Item 3, “Someone to give you good advice about a crisis” was changed to “Someone to give you good advice if it was found that something was wrong in your mammogram.” Item 4, “Someone to confide in or talk to about yourself or your problems” was changed to “Confide in or talk about how to go about getting a mammogram.”

Item 6, “Someone to share your most private worries and fears with” was changed to “Someone to share your worries about getting a mammogram because you might find out something is wrong.”

Item 10, “Someone to take you to the doctor if you needed it” was changed to “Someone who accompanies you to get a mammogram if necessary.”

Item 11, “Someone to prepare your meals if you were unable to do it yourself.” was changed to “If you need to spend a lot of time to get a mammogram and have someone to do your house chores on that day.”

Item 14, “Someone to love and make you feel wanted” was changed to “Have someone who loves you and makes you feel valued.” Similar to the original MOS-SSS, the pilot test resulted in no deletion of items, and the Farsi version contained 19 items from the MSS.

### Pilot study of validation

The preliminary Farsi version of the MSS was pre tested with 29 Iranian women who were recruited in health care centers to evaluate if the questions were unambiguous and easily understood. The inclusion criteria for the pilot study considered women aged 40 or older, not having breast cancer, were not pregnant or breastfeeding, and were able to read and write Farsi.

### Sample validation

The participants were recruited from women referring to health care centers in Sanandaj, Iran. Among 23 health care centers, 12 centers were selected by cluster random sampling. Then, a range of 33 to 36 women was randomly selected for each of the centers. Following ethics approval from the Ethical Committee of Kurdistan University of Medical Sciences, 485 questionnaires were sent to eligible women along with an explanatory letter and an informed consent form. For this sample, eligibility criteria were the same as the pilot test except the criteria for screening was having had at least 1 mammogram in the past two years. A total of 441 questionnaires (91%) were completed. Of this number, 434 participants were divided into two samples based on the time of entry into the study by the participants. Sample 1 (*N* = 204) data were used for exploratory factor analysis (EFA). Sample 2 (*N* = 230) data were used for cross-validation of the confirmatory model derived from Sample 1 data. Statistical tests, including the t-test for continuous variables and chi-square test for categorical variables, were used to compare and contrast key demographic variables. After Confirmatory Factor Analysis (CFA), by using a convenience sampling method, two groups of Iranian women (*n* = 30, *n* = 48) were referred to health care centers having the same eligibility criteria as the pilot study and were recruited for assessing internal consistency using Cronbach’s alpha coefficient and test-retest reliability.

### Statistical analysis

Chi-square tests were used to identify the effect of socio-demographic and mammography social support on adherence to screening mammography. The study investigated the suitability of the respondent data for factor analysis through the Kaiser-Meyer-Olkin (KMO). The used extraction method in factor analysis included the Principal component analysis with Varimax rotation where there were a loading criteria of 0.4 or more. The used fit indices included Comparative Fit Index (CFI), Tucker-Lewis Index (TLI), Root Mean Square Error of Approximation (RMSEA), and Standardized Root Mean Square Residual (SRMSR). Cut-off points for inferring adequate fit indices were set at (CFI > 0.95; TLI > 0.95; Root Mean Square Error of Approximation (RMSEA), and Standardized Root Mean Square Residual (SRMSR) with acceptable values of zero to one.

All analyses were conducted using STATA Version 13. In addition to conducting CFA on the social support scale. The internal consistency was evaluated by: (1) the correlation between the individual items and the corrected-item total score; (2) alpha coefficient; and (3) if an item deleted from the scale, Cronbach’s alpha would not increase more than 0.10 [46]. The stability of MSS scores was estimated by calculating test-retest reliability over time a 2-week interval. In addition, we conduct discriminant validity through binary logistic regression. To this done, mammography adherence as the outcome variable classified into two categories: 0 representing not having one mammogram in the 2 years ago and 1 representing having at least one or two or more mammograms in the before 2 years. The exposure variables included the total score of perceived social support and related subscales.

## Results

### Demographic characteristics

Study sample 1 included 204 participants for the EFA. Construct validity was determined by CFA using study sample 2 comprised of 230 women. Table 1 provides the descriptive characteristics of the sample. The mean participants age was 48.12 ± 8.91, distributor ranging from 40 to 67 years, with 76% married women and 65% homemakers. More than half of participants had not received the equivalent of a high school diploma (53%). The health insurance covered most participants (91%). Approximately one-third of the sample (*N* = 141) recieved had at least one mammogram in the past 2 years. The prevalence history of breast cancer in their family or having a history of any type of past breast problems were (2.9%) and (12%) respectively.

**Table 1:** Characteristics of Study Participants (*N* = 434)

Characteristics	*N* (%)
Age	
40-45	220 (50.7)
46-50	100 (23.1)
51-55	65 (15.0)
56-60	32 (7.0)
61 and older	17 (4.0)
Marital status	
Single	25 (5.8)
Married	332 (76.4)
Widowed	77 (17.8)
Education status	
Primary	110 (25.3)
Secondary	120 (27.7)
Diploma	97 (22.3)
Academic	107 (24.7)
Employment status	
Homemaker	281 (64.8)
Employed	153 (35.4)
Menopause	
Yes	121 (27.8)
No	313 (72.2)
History of personal breast problem	
Yes	52 (12.0)
No	382 (88.0)
Family history of breast cancer	
Yes	13 (2.9)
No	421 (97.1)
Health insurance	
Yes	396 (91.2(
No	38 (8.8)
History of having mammography in past 2 y	
Yes	141 (32.4)
No	293 (67.6)

### Construct validity

#### Exploratory factor analysis, Farsi version of mammography social support scale

The Kaiser-Meyer-Olkin measure (KMO) was .88, and the Bartlett’s test of sphericity was significant (*x*^*2*^ = 2540.17, df = 171, *P* < 0.001), indicating the data meet quality for factor analysis. The EFA produced a result indicating that the 19-item social support for mammography scale resulted in 3 factors with eigenvalues more than 1, explained 66% of the variance. The standardized factor loading of the 19 items, all eigenvalues in a factor and the percentage of explained variance are presented in Table 2. No cross-loading was observed, and all 19 items loaded higher than the set threshold of 0.40 for inclusion in the interpretation of factors.

**Table 2:** Factor loadings, item analysis, and the item total correlations for the 19 items in the Farsi Version of the Mammography Social Support Scale (*N* = 204)

**Mammography Social Support**	Factor loading Factor 1	Factor loading Factor 2	Factor loading Factor 3	Item Mean (SD)	Corrected Item/Total Correlation	α if Item Deleted
1. Someone you can count on to listen to you when you need to talk (EIS 1)	0.415	**0.452**	0.081	3.03 (1.21)	.44	.85
2. Someone to give you information to help understand mammogram procedure (EIS 2)	0.090	**0.814**	0.138	2.61 (1.18)	.55	.83
3. Someone to give good advice if find out something is wrong in your mammogram (EIS 3)	0.049	**0.837**	0.164	2.44 (1.21)	.62	.83
4. Someone to confide in or talk how to go about getting a mammogram (EIS 4)	0.080	**0.831**	0.169	2.83 (1.28)	.68	.82
5. Someone whose advice you really want (EIS 5)	0.269	**0.665**	0.098	3.11 (1.19)	.59	.83
6. Someone to share your worries about getting a mammogram because you might find out something is wrong (EIS 6)	0.278	**0.783**	0.209	2.91 (1.20)	.65	.82
7. Someone to turn to for suggestions about how to deal with a personal problem (EIS 7)	0.439	**0.631**	0.244	2.93 (1.12)	.62	.83
8. Someone who understands your problems (EIS 8)	0.507	**0.585**	0.243	3.13 (1.20)	.58	.83
9. Help you if confined to bed (TS 1)	0.456	0.287	**0.550**	3.67 (1.29)	.55	.79
10 Someone who accompanies you to get a mammogram if necessary (TS 2)	0.106	0126	**0.774**	3.08 (1.28)	.46	.82
11. If you need to spend a lot of time getting a mammogram and need someone to do your house chores on that day (TS 3)	0.359	0.225	**0.773**	3.19 (1.43)	.47	.68
12- Someone to help with daily chores (TS 4)	0.420	0.244	**0.748**	3.17 (1.36)	.53	.70
13. Someone who shows you love and affection (AS 1)	**0.645**	0.250	0.358	3.68 (1.09)	.69	.70
14. Someone who loves you and makes you feel valued (AS 2)	**0.46**	0.069	0.248	3.51 (1.15)	.62	.77
15. Someone who hugs you (AS 3)	**0.667**	0.170	0.420	3.67 (1.21)	.65	.74
16. Someone to do something enjoyable with (POS 1)	**0.790**	0.214	0.310	3.73 (1.28)	.48	.82
17. Someone to get together with for relaxation (POS 2)	**0.683**	0.127	0.008	3.24 (1.29)	.61	.88
18. Someone to love and make you feel wanted (POS 3)	**0.794**	0.185	0.254	3.46 (1.26)	.55	.83
19. Someone to do things with to help you get your mind off things (POS 4)	**0.775**	0.195	0.383	3.58 (1.20)	.58	.82
Eigenvalue	7.50	2.58	1.30			
Variance (%)	39.49	13.58	6.84			
Total variance (%)	65.62					
Scale mean (SD)	60.99 (14.61)					

Bolded indicates highest factor loadings.

*Abbreviation: EIS* Emotional/informational support, *TS* tangible support, *AS* affectionate support, *POS* positive social interaction

#### Confirmatory factor analysis, Farsi version of the mammography social support scale

**Fig. 1:**
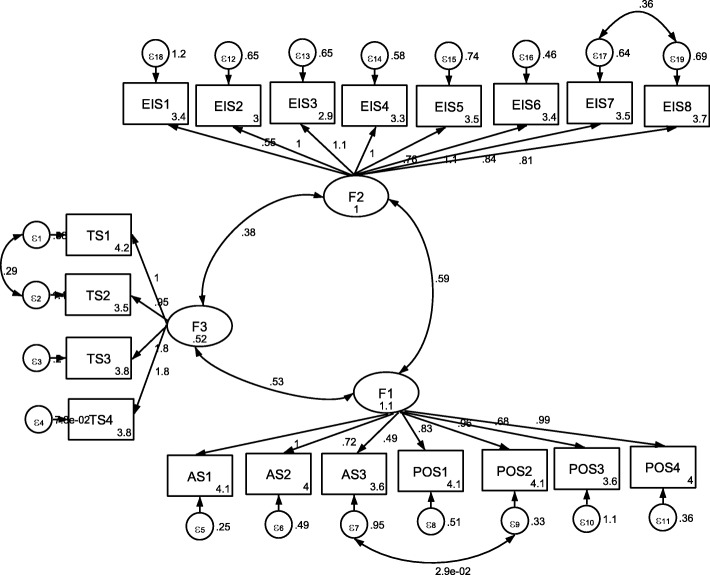
Standardized solution for the revised model based on confirmatory factor analysis mammography self-efficacy. Numbers circles in rectangles indicate measurement errors

The measurement model was not an optimal model. The overall fitting results of confirmatory factor analysis with 19 items were: *x*^2^ = 813.69, CFI = 0.85, TLI = 0.83, RMSEA = 0.11 and SRMSR = 0.08, showed the model’s unsatisfactory fitting based on adequacy criteria. A new confirmatory factor analysis considered adding a correlated error term between items (AS 2) and (POS 1) on factor 1, affectionate support and positive social interaction related to the perception of having people who make one feel socially significant, emotional exchanges and pleasure with one’s social life. The results of the modification also advocated to being a correlated errors on items (EIS 7) and (EIS 8) to reflect the individual’s perception of the availability of support from her social network, sharing emotional aspects that help her to face problems on factor 2 as well on items (TS 1) and (TS 2) on factor 3 related to perception of social support to help in coping with concrete problem situations. These modifications resulted in a good fit to the data: *×* ^2^ = 299.63, CFI = 0.96, TLI = 0.95, RMSEA = 0.06 and SRMSR = 0.04 (Table 3; Fig. 1).

**Table 3:** The Fit Indexes of the Initial and Revised Model of the Confirmatory Factor Analyses for Farsi Version of the Mammography Social Support Scale (*N* = 230)

	Indexes Values						
	*x* ^2^	df	*x*^2^/df	CFI	TLI	RMSEA	SRMSR
Initial model	813.98	150.73	5.4	0.85	0.83	0.11	0.08
Revised model	299.63	130.27	2.3	0.96	0.95	0.06	0.04

*Abbreviations: CFI* Comparative Fit Index, *RMSEA* Root Mean Square Error of Approximation, *SRMSR* Standardized Root Mean Square Residual, *TLI* Tucker Lewis Index

### Reliability

#### Measurement internal consistency and item-total correlation

The scale was found to be internally reliable with a corrected item-total correlation ranging from 0.44 to 0.69, which means that the items were sufficiently related and contributed to scoring measurement. After factor structure confirmation, the Cronbach’s alpha coefficient for the total scale was 0.91, indicating excellent internal consistency. The subscale alpha coefficients were 0.85, 0.82 and 0.90 for emotional/informational, tangible, affectionate and positive social interaction social support respectively.

#### Stability

Test-retest reliability was conducted after two weeks with a random sub-sample of 48 women (response rate 96%), and the correlation coefficients for MSS was 0.95 (*p* < 0.001), indicating high stability.

#### Testing discriminant validity

For the results of discriminant validity in terms of mammography adherence, the women in the sample differed significantly by employment status (*x*^2^ = 5.35, *df* = 1, *p* < .02), history of breast problems (*x*^2^ = 29. 90, *df* = 1, *p* < .001), family history of breast cancer (*x*^2^ = 10.15, *df* = 1, *p* < .001), and health insurance (*x*^2^ = 5.58, *df =* 1, *p* < .01). The discriminant validity results demonstrated those who had higher scores on social support and its related subscales were more likely for having a history of receiveing a mammography in the past 2 years. Women with greater total social support score were more likely to repeat mammography than who with lower social support scores (OR, 0.92; 95% CI, 0.91–0.95; *P* < 0.001). Women with higher values on the three subscales emotional/informational, affectionate and positive social interaction repeated mammography more than women with lower values on the three (ORs, 0.89; 0.92 and 0.87 respectively) and (*p* < .03–.001) (data not shown).

## Discussion

While, measuring the functional components of social support for many behaviors have been conducted for several diseases [43, 47, 48], this study provides the first validation of a social support instrument of mammography screening in an Iranian population. It has long been acknowledged that social support facilitates health behavior change as an important resource [18, 20, 34]. However, the construct is not routinely examined as affecting breast cancer screening behaviors and might be an area for intervention.

The Farsi version of the MSS produced satisfactory psychometric properties. The results of the reliability analysis showed that the MSS was reliable and it could be used among Iranian women for measuring social support specific to mammography screening behavior. The Farsi version of the social support for mammography screening scale consisted of 19 items and resulted in three conceptual dimensions. These dimensions correspond to mammography screening social support as it has been defined and used in this study.

The resulting three-factor solution produced in the current study is different from the original version [15] that showed four factors (emotional/informational, tangible, affectionate, and positive social interaction), were sufficient to understand social support among chronically ill patients. A potential reason is that different aspects of social support are relevant to cancer screening behaviors [3, 20, 21] than chronic disease management.

Consistent with the study by Sherbourne [15], all of the items in each of the subscales, emotional/informational support and tangible support loaded on their respective factors except the affection and positive social interaction subscales exhibited factor loadings on the single factor (factor 1). Similar results were identified in the process of validating the Brazilian Portuguese version of the social support scale among Brazilian Hodgkin’s lymphoma survivors [43], as well the Brazilian version among the general population [47]. Our results showed high correlation coefficients between items from affection and positive social interaction dimensions. Our results do not fully support the dimensionality of the original instrument [15]. The positive social interaction support and emotional support items failed to separate from each other. Therefore, these items might be highly correlated [3, 20]. A likely explanation for the lack of distinction between positive social interaction and affectionate support could be related to health outcomes [15] and is consistent with previous research [20, 43, 47–49]. The lack of distinction between these two constructs might be related to the nature of the sample, recommended behavior, culture, and other socioeconomic factors. Future research needs to be conducted to corroborate our findings.

The MSS scale distinguished women by screening status according to their mammography social support scores. The consistency of the results of this study and those of previous studies [3, 17, 20, 34], would contribute more support for the feasibility of MSS in this poorly studied population.

The standardized factor loading was acceptable (0.45 to 0.83) on social support for the mammography scale items and were moderate to high, showing that most of the relevant observed variables are sufficiently measured by the latent construct of mammography screening social support. These results indicated the comparability our findings with those which reported three factors in a validation study of social support among Brazilian Hodgkin’s lymphoma survivors where factor loadings were within 0.42–0.84 and the Brazilian version of the social support scale among general population produced factor loadings ranges within 0.41–0.91 [43, 47]. These results are different from those reported in Sherburne’s study where factor loadings were within 0.72–0.90 [15], and developed to apply to chronically ill patients and produced four factors.

Consistent with earlier versions reported for MOS-SSS (reference) and MOS (reference) the Cronbach’s Alpha results for the Farsi version of the MSS demonstrated excellent reliability (0.91). In Soares’s and Griep’s studies, in which the MOS-SSS included 19 items, the Cronbach’s alpha values were 0.95, and 0.81, respectively [43, 47]. Sherburne et al., reported Cronbach’s alpha of 0.97 for the MOS [15]. Overall, measurement of stability the MSS and three of its subscales supported by the results of test-retest analysis over a 2-week period (*r* = .95).

Because the EFA finding did not offer strong support for the construct validity of the MSS, we used CFA to examine if the hypothesized model identified from EFA fit the data. Therefore, we used CFA to examine whether the hypothesized model identified from EFA fit the data. We then modified the model consequently. The CFA results proceeded to support the three factors of the MSS. While the primary fit indices were not fully endorsed for this model, a modified three-factor correlated model resulted in better-fit indices.

Changes in wording suggested by the expert panel to capture the concept of social influence and the theoretical attributes of social support as defined by Sherbourne helped to refine the scale [15]. The items addressed potential different sources “to give information related mammogram procedure (EIS 2),” “talk about how to go about getting a mammogram (EIS 4),” and “give good advice to find out if something is wrong in your mammogram” (EIS 5) explored moderate to strong correlation (0.66–0.81) with emotional/informational support dimension. These items evaluated the perception of people for whom the individual could rely on to obtain information about obtaining a mammography. Psychosocial barriers to receiving mammography screening include worry, pain, shame, feelings of being too old to be screened, and fear [50]. Overcoming these barriers consequently may affect the extent to which someone relied on others’ support [3, 20]. Furthermore, overcoming these barriers is more important in Iran where there is no national breast cancer screening program [8]. As in therefore, it is expected perceived social support be adapted to a specific area of performance which appraises how much persons acknowledge that they are supported when having trouble during their lives. Material social support reflects access to someone who can help when having a problem [15]. Similarly, in confrontation with problem situations the perception of social support might help to overcome the problems.

Those modifications recommended by the expert panel related to “having someone accompany you to get a mammogram (TS 2)” “help to you to do your house chores” (TS 3) and “doing daily chores more important than getting a mammogram” (TS 4) showed high correlation coefficients (074–.077) on the dimension of tangible support. Such a construct allows the possibly to assess the amount of each dimension might impacts a person’s decision to receive a mammogram. Some limitations need to be considered. First, there was likely some non-response bias given only 26% of the overall sample was 50 years of age and eligible for mammogram screening according to the recommended screening age for mammography in Iran. Another limitation of this study is that findings may not be generalizable to all breast cancer screening behaviors such as a clinical breast exam. Mammography screening adherence has different definitions such as first-time screening or repeat mammography.

## Conclusion

Breast cancer screening behavior is a complex concept. As more attention is given to breast cancer screening rates among Iranian women, health promotion researchers can promote this practice by better understanding women’s social support for breast cancer screening practices. The mammography social support scale adapted for this study can be used to assess the role of cross-cultural differences in adaptation and validation of MSS. More specifically, this research helps to identify the kinds of support available and personal relationships relevant to mammography screening behavior in Iranian women. The differences in the strength of association of social support on mammography screening behavior reflected the range of concepts of social function. Since the different languages, cultures, and health care systems, could influence on the perception of social support for mammography, testing the MSS is most recommended in other populations, and methods of promotion for breast cancer screening programs. Future research should examine the factor structure and internal consistency of the MSS among diverse populations.

## Abbreviations

BSE: Breast Self-Exam
CBE: Clinical Breast Examination
CFI: Comparative Fit Index
CVI: Content Validity Index
KMO: Kaiser-Meyer-Olkin
MSS: Mammography Social Support
PSI: Positive Social Interaction
RMSEA: Mean Square Error of Approximation
TLI: Tucker Lewis Index
